# Person-centered support for patients with a pituitary tumor following surgery

**DOI:** 10.1530/EC-24-0686

**Published:** 2025-01-28

**Authors:** Sofie Jakobsson, Oskar Ragnarsson, Tobias Hallén, David Krabbe, Ann-Charlotte Olofsson, Daniel S Olsson, Penelope Trimpou, Thomas Skoglund, Gudmundur Johannsson

**Affiliations:** ^1^ Institute of Health and Care Sciences, Sahlgrenska Academy, University of Gothenburg, Gothenburg, Sweden; ^2^ University of Gothenburg Centre for Person-Centred Care (GPCC), Sahlgrenska Academy, University of Gothenburg, Gothenburg, Sweden; ^3^ Region Västra Götaland, Sahlgrenska University Hospital, Department of Endocrinology, Gothenburg, Sweden; ^4^ Department of Internal Medicine and Clinical Nutrition, Institute of Medicine, Sahlgrenska Academy, University of Gothenburg, Gothenburg, Sweden; ^5^ Wallenberg Center for Molecular and Translational Medicine, Institution of Medicine at Sahlgrenska Academy, University of Gothenburg, Gothenburg, Sweden; ^6^ Region Västra Götaland, Sahlgrenska University Hospital, Department of Neurosurgery, Gothenburg, Sweden; ^7^ Department of Clinical Neuroscience, Institute of Neuroscience and Physiology, Sahlgrenska Academy, University of Gothenburg, Gothenburg, Sweden; ^8^ Region Västra Götaland, Sahlgrenska University Hospital, Department of Rehabilitation Medicine, Gothenburg, Sweden; ^9^ Late-stage Clinical Development, Cardiovascular, Renal and Metabolism (CVRM), BioPharmaceuticals R&D, AstraZeneca, Gothenburg, Sweden

**Keywords:** pituitary tumor, non-functioning pituitary adenoma, person-centered care, surgery

## Abstract

**Objectives:**

To evaluate whether a person-centered care practice following surgery for pituitary tumors increased psychological well-being. Secondary aims were to study whether person-centered care would lead to better health status, less fatigue and better self-efficacy.

**Design and methods:**

This study is a prospective, single-center study using a quasi-experimental design to evaluate the effect of a 12-month person-centered practice by means of a name-given nurse care manager, an interdisciplinary team and peer support against usual care. All patients (≥18 years) with a benign pituitary tumor and planned for endoscopic transsphenoidal surgery were consecutively invited to participate. Psychological well-being, self-reported health, fatigue and self-efficacy were assessed before surgery, at discharge and 3–6 and 12 months after surgery.

**Results:**

In total, 86 patients in the intervention group and 68 patients in the control group were included. Psychological well-being improved 12 months following surgery in both groups to comparable levels. The intervention group had a greater improvement in anxiety compared to the control group (*P* = 0.02). No differences were seen between groups in self-reported health status, fatigue or self-efficacy. Patients in the intervention group with other types of pituitary tumors than non-functioning pituitary adenomas showed a greater improvement in psychological well-being than in the control group.

**Conclusion:**

Our intervention did not result in major advantages in terms of health or psychological well-being. The study does, however, suggest that the intervention may reduce anxiety 12 months after surgery and that certain subgroups of patients may benefit more from a structured person-centered practice following pituitary surgery.

## Introduction

Pituitary tumors are mostly benign tumors that may occur at any age but most often in persons between 40 and 50 years of age at the peak of their professional careers (1). The most common pituitary tumors are non-functioning pituitary adenomas (NFPAs). The annual incidence of clinically significant pituitary adenomas is approximately 4.0 per 100,000 inhabitants (1). Although these tumors are histologically benign, the tumor itself and its treatments can lead to lifelong consequences affecting a person’s health and life expectancy. The disease and its treatment necessitate long-term contact with specialized healthcare providers to manage a complex endocrine replacement therapy and metabolic care, to monitor tumor remnants and to receive support due to cognitive deficits and distressing physical and psychological symptoms (2).

Pituitary tumors pose a significant challenge for patients as they may impact many aspects of their lives. Symptoms such as fatigue, impaired memory and concentration, anxiety, sleep disturbances and sexual dysfunction affect health to various degrees (3, 4, 5, 6, 7). The time period around diagnosis and surgery can raise physical and cognitive issues as well as emotional and existential concerns, implying a need for extensive disease-related information (8, 9). The post-surgery recovery period involves a return to normal daily activities, yet there may persist a lingering concern about the possibility of tumor recurrence, the realization of lifelong endocrine replacement therapy and the persistence of long-term symptoms (8).

Person-centered care aims to be respectful of how patients view their health situation and what are their needs as well as their resources and preferences (10, 11). A collaborative relationship between healthcare professionals and patients is emphasized with the main goal of enhanced health for each patient through narration, partnership and documented healthcare planning (11). Several studies evaluating person-centered care across various chronic diseases have shown beneficial effects on self-reported outcomes, such as increased self-efficacy, improved satisfaction of care, symptom alleviation and improved activities of daily life (12). Evaluation of person-centered care for patients with pituitary tumors is limited. A self-management education program for patients with pituitary tumors increased their self-efficacy and, to some extent, improved mood (13). An intervention designed specifically for patients with Cushing’s syndrome showed an improvement in health outcomes through a nurse-led educational program (14).

We hypothesized that patients with pituitary tumors who received extended person-centered support after pituitary surgery would report better health than patients given usual care. The primary aim of this prospective intervention study was to evaluate whether support within a person-centered care practice over a 12-month time period after pituitary surgery would increase psychological well-being. Secondary aims were to evaluate whether the addition of person-centered care would result in better self-reported health status, less fatigue and higher self-efficacy 12 months after surgery.

## Methods

### Design

This was a prospective, single-center, quasi-experimental study with a control group. For more detailed information on the study design and methodology, see the published study protocol (15), which adheres to the CONSORT statement and is registered at https://www.researchweb.org/is/sverige/project/161671.

The study was carried out in two sequential steps: i) a control group of patients who received standard care for 12 months after pituitary surgery, followed by ii) an intervention group in which patients received extended support within a person-centered practice for 12 months after surgery.

The study was conducted in accordance with the Declaration of Helsinki.

### Participants

All patients >18 years of age diagnosed with a benign pituitary tumor and planned for neurosurgery using an endoscopic transsphenoidal technique at a single study center were consecutively invited to participate in the study before their surgery. Exclusion criteria were compromised health conditions that might restrict understanding of the study or ability to adhere to the study protocol (e.g., cognitive impairments or drug addiction). The power calculation was based on two previous endocrine replacement therapy interventions evaluating improvement in psychological general well-being (PGWB) (16, 17). The power calculation estimated that 90 patients should be included in each group, considering a 10% discontinuation rate.

### Intervention

The goal of the person-centered practice for patients with a pituitary tumor was to integrate efficiently organized and evidence-based medical care with nurse-led support based on each patient’s experiences and a personal health plan. Before the intervention was launched, two focus groups of patients with long-term experience of having a pituitary tumor were formed and used to contribute information to the design of the planned intervention. The focus groups contributed to identifying issues and perceived limitations surrounding current care and specific patient needs and discussions on what components would contribute to care in line with a person-centered practice. A comprehensive description of both standard care and the intervention has been published elsewhere (15). All patients, in both the control group and intervention group, received standard of care for both hospital-based inpatient and outpatient care. Specifically, in the first 24 h after surgery, patients are monitored in an inpatient unit with a focus on neurological status and fluid and electrolyte balance. After this initial period, they are monitored for an additional 5 days in the inpatient endocrinology department. Before discharge on postoperative day 6, their endocrine function is evaluated. Four to five weeks after the inpatient care, a postoperative assessment is performed by an endocrinologist where hormonal status is reassessed, and any complications are documented. Subsequent outpatient visits with a physician and a nurse are scheduled based on tumor type, degree of hypopituitarism and reported symptoms.

The intervention was developed and accomplished based on principles of person-centered care, which in practice entails a focus on the narrative from patients and significant others, a focus on establishing and working in a partnership between patient, significant others and the care team and finally ongoing care documentation in a health plan (11). In addition to the standard care described above, all patients in the intervention group were assigned a nurse care manager to secure continuously available support after surgery. The nurse and the patient had first contact at the inpatient unit before discharge after surgery. At this time point, patient-held documentation, a health book, was initiated and a health plan with mutually agreed goals was formed based on the patient narrative. During the 12-month intervention period, patients had continuous access to the nurse care manager by telephone, and each patient had planned individual meetings with their nurse care manager. More than 93% of the patients received the planned, structured, nurse-led follow-up at each of the four time points specified during the study period. There was no pre-existing manual dictating the content of these conversations. The primary focus of the follow-up was to ensure that the patient could share their story, acquire understanding about their condition, receive assistance for self-care and be empowered to utilize their own resources in managing treatment and symptoms. This approach aimed at resulting in an updated health plan at each subsequent follow-up.

An education program, which also was a part of the intervention but not included in standard care, reached 42 of the 100 included patients and 14 of their significant others. The one-time session education program aimed to enhance patient knowledge of the disease and treatment by meeting a multiprofessional team of neurosurgeons, endocrinologists and nurse care managers and by supportive interaction among peers.

### Data collection

The PGWB scale, a 22-item questionnaire comprising six domains (anxiety, depression, positive well-being, self-control, general health and vitality), was used to assess the primary outcome of self-perceived psychological well-being (18). The questionnaire uses Likert subscales for each item with the following scores: anxiety (5–30), depression (3–18), positive well-being (4–24), self-control (3–18), general health (3–18) and vitality (4–24), with higher scores indicating better psychological well-being. The total PGWB score range is 22–132, where 132 points represent excellent psychological well-being. Self-reported health status was assessed using the EuroQoL 5-Dimension 5-Level (EQ-5D-5L) scale, which comprises a visual analog scale from 0 (worst health) to 100 (best health) for five dimensions of health (mobility, self-care, usual activities, pain/discomfort and anxiety/depression) (19). Patients also completed the Multidimensional Fatigue Inventory-20 (MFI-20), where five subscales (general fatigue, physical fatigue, reduced activity, reduced motivation and mental fatigue) were each assessed by four questions using 5-point Likert scales (1–5) (total range for each subscale: 4–20), with higher scores indicating more fatigue (20). The self-assessed generalized self-efficacy (GSE) scale measures how the patient perceived their possibility of adhering to the goals being set and find solutions to unforeseen or surprising situations and challenges in life (21). It measures 10 items on 4-point Likert scales (1–4) (total range: 10–40), with higher scores indicating more self-efficacy. Assessments of self-reported data were performed at four time points: before surgery (baseline), at 0 months (discharge) and 3–6 and 12 months after surgery; the time points were chosen based on the clinical care pathway of standard care.

### Data analysis

Categorical data are presented as numbers and percentages, and continuous data are presented as mean, standard deviation, median and range depending on the data distribution. Analysis of covariance was used to adjust the analyses for baseline levels and for other covariates, presenting adjusted least square means (LSMs), standard errors of the mean and 95% confidence intervals (CIs) of the mean along with the difference between the adjusted LSMs with 95% CIs. All tests are two-sided with alpha 0.05. All analyses were performed using SAS 9.4 (SAS Institute Inc., USA).

## Results

**Figure 1:**
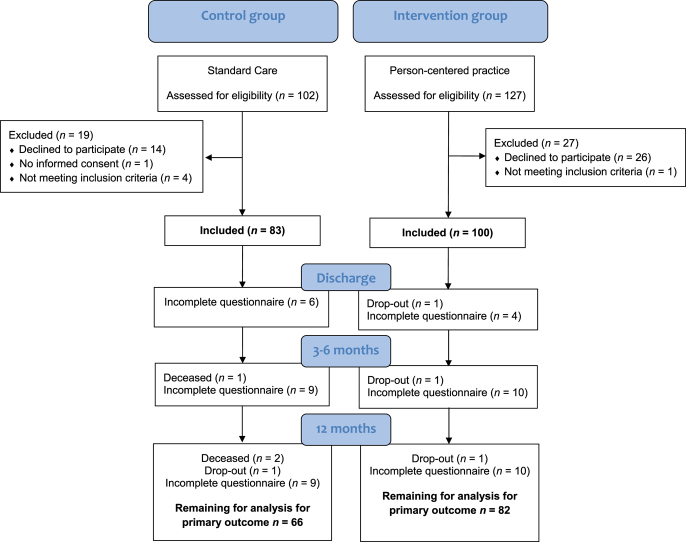
Flow chart for patient recruitment.

A flow chart for patients in the control and intervention groups is shown in Fig. 1. A total of 102 patients were assessed for eligibility in the control group during an enrollment period of 2 years and 3 months, of whom 83 were included to receive standard care. The enrollment period for the intervention group was 3 years and 2 months, assessing 127 patients for eligibility, of whom 100 were included to receive the structured person-centered practice. Demographic factors and clinical data were similar between the two groups at baseline (Supplementary Table S1, see section on Supplementary materials given at the end of the article). The dropout (including deaths) rate was low. The number of evaluable patients reduced with time, primarily due to incomplete questionnaire data (range: 4–11% at the different time points). As a result, the number of patients available for the assessment of change in the primary outcome variable was reduced. The characteristics of patients available for the assessment of change in the self-reported primary outcome variable between baseline and 12 months after surgery are summarized in Table 1. The majority of the patients in the intervention group underwent surgery and were followed up during the COVID-19 pandemic. In total, 39% of the patients in the intervention group received the 12-month intervention before and 61% during the pandemic.

**Table 1:** Patient characteristics and clinical data in the control group receiving standard care and the intervention group receiving person-centered practice.

	Control group (*n* = 68)	Intervention group (*n* = 86)
Gender, *n* (%)		
Women	32 (47)	33 (38)
Men	36 (53)	53 (62)
Age, median (range), years	59 (19–83)	62 (24–87)
Living arrangements, *n* (%)		
Cohabitating	52 (77)	63 (75)
Living alone	16 (23)	21 (25)
Country of birth, *n* (%)		
Sweden	58 (85)	74 (88)
Outside Sweden	10 (15)	10 (12)
Education, *n* (%)		
Elementary school	13 (19)	11(13)
High school	34 (50)	45 (54)
University	21 (31)	28 (33)
Occupation, *n* (%)		
Working/studying	32 (48)	43 (51)
Other	34 (52)	41 (49)
Diagnosis, *n* (%)		
NFPA	43 (63)	66 (77)
Cushing’s disease^§^	5 (7)	4 (5)
Acromegaly^║^	9 (13)	9 (10)
Thyrotropinoma	1 (1)	1 (1)
Prolactinoma	2 (3)	0
Craniopharyngioma	2 (3)	2 (2)
Rathke’s cleft cyst	4 (6)	2 (2)
Others*	2 (3)	2 (2)
Cortisol insufficiency,^†^ *n* (%)		
Yes	25 (37)	28 (33)
No	43 (63)	58 (67)
Prior surgery, *n* (%)		
Yes	12 (18)	12 (14)
No	56 (82)	74 (86)
Duration of hospitalization,^‡^ mean (SD) [range], days	7.61 (3.5) [4–30]	7.87 (2.2) [4–15]

Abbreviation: NFPA, non-functioning pituitary adenoma.

* Includes chondroid chordoma, hypophysitis, pituitary cytoma and unclear (each *n* = 1).

^†^ Post-surgery.

^‡^ Inpatient care after surgery.

^§^ Biochemical control was achieved in all five patients in the control group and in four out of five patients in the interventional group.

^║^ Biochemical control was achieved in six out of nine patients in the control group and in eight out of nine patients in the interventional group.

### **Psychological general **w**ell-**b**eing score**

**Figure 2:**
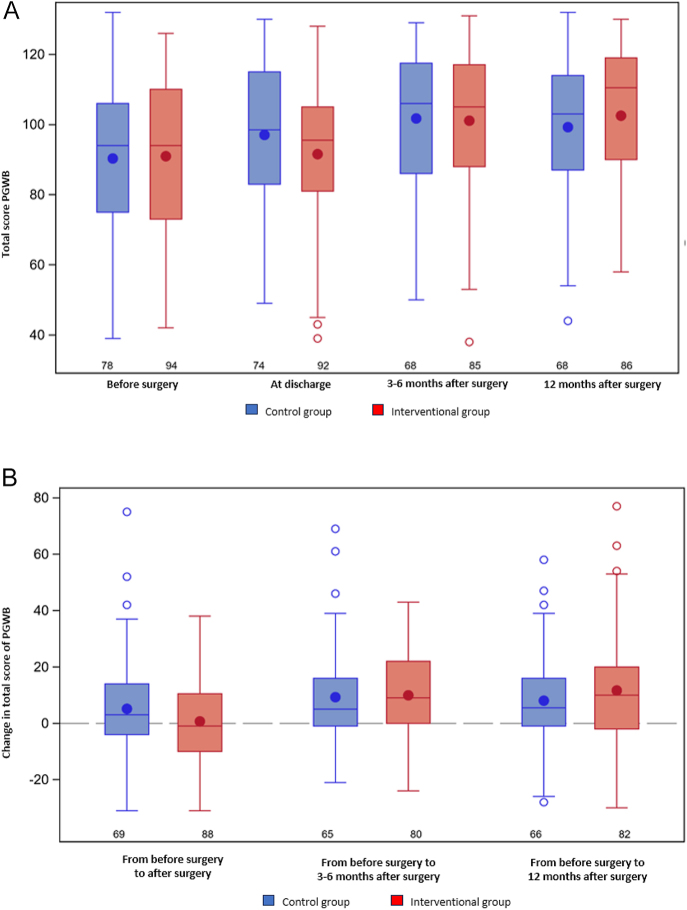
Whisker plots showing (A) median and interquartile range of total PGWB score in the intervention and control groups at baseline (before surgery) and different time points over 1-year follow-up and (B) change in total PGWB score between the intervention group and the control group at the different time points over 1-year follow-up. There were no significant differences between the groups at any time point. Total scores range from 22 to 132. Abbreviation: PGWB, psychological general well-being.

The primary study endpoint, total PGWB score, increased over 12 months in both the control and intervention groups (Fig. 2); however, there was no statistically significant difference between the groups (Supplementary Table S2). Of the six PGWB subscales measuring affective states, there was a significantly greater improvement at 12 months compared to baseline for anxiety in the intervention group versus the control group: the adjusted mean difference between the groups was −1.6 (95% CI: −2.9 to −0.2; *P* = 0.02). There were no significant differences between the groups for changes in the other PGWB subscales (depression, positive well-being, self-control, general health and vitality) during the 12 months after surgery (Table 2).

**Table 2:** Adjusted and unadjusted differences in PGWB total and subscale scores from baseline to 12 months.

PGWB total or subscale scores	Control group (*n* = 68)		Intervention group (*n* = 86)		Adjusted* mean difference between groups (95%CI) [significance]
	Mean (SD) [range]	Adjusted* mean (SEM) [95% CI]	Mean (SD) [range]	Adjusted* mean (SEM) [95% CI]	
Total	8.0 (16.4) [−28 to 58]	8.1 (1.9) [4.4 to 11.8]	11.6 (18.7) [−30 to 77]	11.6 (1.7) [8.2–14.9]	−3.4 (−8.5 to 1.6) [*P* = 0.18]
Subscale					
Anxiety	1.9 (5.2) [−9 to 15]	2.1 (0.5) [1.1–3.0]	3.7 (5.1) [−5 to 21]	3.6 (0.4) [2.7–4.5]	−1.6 (−2.9 to −0.2) [*P* = 0.02]
Depression	0.8 (2.7) [−5 to 9]	0.9 (0.3) [0.3–1.4]	1.3 (2.7) [−5 to 13]	1.2 (0.2) [0.7–1.7]	−0.4 (−1.1 to 0.4) [*P* = 0.33]
Positive well-being	1.5 (3.3) [−8 to 8]	1.5 (0.4) [0.7–2.3]	1.5 (4.0) [−10 to 12]	1.6 (0.4) [0.9–2.3]	−0.1 (−1.2 to 1.0) [*P* = 0.86]
Self-control	0.8 (2.9) [−5 to 11]	0.8 (0.3) [0.3–1.3]	1.1 (2.7) [−5 to 10]	1.1 (0.2) [0.6–1.6]	−0.3 (−1.0 to 0.5) [*P* = 0.48]
General health	1.1 (3.0) [−5 to 11]	1.2 (0.3) [0.5–1.8]	1.8 (3.2) [−4 to 13]	1.8 (0.3) [1.2–2.4]	−0.7 (−1.6 to 0.2) [*P* = 0.15]
Vitality	1.6 (3.8) [−8 to 12]	1.6 (0.5) [0.7–2.6]	1.9 (4.8) [−12 to 14]	1.9 (0.4) [1.1–2.7]	−0.2 (−1.5 to 1.0) [*P* = 0.67]

Abbreviations: CI, confidence interval; PGWB, psychological general well-being; SD, standard deviation; SEM, standard error of the mean.

* Adjusted for age, gender, education and cortisol insufficiency using analysis of covariance (ANCOVA).

In a patient subgroup analysis of total PGWB score, those in the intervention group with pituitary tumors other than the NFPA (*n* = 20) had a greater improvement between baseline and 12 months than the control group (*n* = 24): the mean adjusted difference between the groups was −14.8 (95% CI: −24.0 to −5.7; *P* = 0.002). In addition, there was a trend for greater improvement in total PGWB score in patients who had first-time surgery in the intervention group (*n* = 71) compared to the control group (*n* = 55): the mean adjusted difference between the groups was −5.2 (95% CI −10.7 to 0.1; *P* = 0.055) (Table 3).

**Table 3:** Adjusted and unadjusted differences in PGWB total scores of psychological well-being from baseline to 12 months in subgroups of patients with pituitary tumors.

Subgroup	Control group (*n* = 68)		Intervention group (*n* = 86)		Adjusted* mean difference between groups (95%CI) [significance]
	Mean (SD) Median (range)	Adjusted* mean (SEM) [95% CI]	Mean (SD) Median (range)	Adjusted* mean (SEM) [95% CI]	
Surgical status					
First surgery (*n* = 55/71^†^)	8.6 (17.1) 5 (−28 to 58)	8.7 (2.0) [4.7–12.7]	14 (18.2) 13 (−22 to 77)	14 (1.8) [10.4–17.5]	−5.2 (−10.6 to 0.1) [*P* = 0.055]
Prior surgery (*n* =11/11^†^)	5.1 (11.8) 9 (−11 to 28)	4.3 (3.9) [−3.9 to 12.6]	−3.8 (14.5) −3 (−30 to 27)	−3.1 (3.9) [−11.3 to 5.2]	7.4 (−4.3 to 19.2) [*P* = 0.020]
Tumor type					
NFPA (*n* = 42/62^†^)	11.9 (17.8) 8.5 (−28 to 58)	11.7 (2.2) [7.3–16.1]	9.6 (17.8) 8.0 (−30 to 77)	9.7 (1.8) [6.1 to 13.3]	1.9 (−3.7 to 7.6) [*P* = 0.050]
Other^‡^ (*n* = 24/20^†^)	1.2 (10.9) 2 (−26 to 26)	2.1 (3) [−4.01 to 8.20]	18 (20.6) 15 (−9 to 63)	16.9 (3.3) [10.2–23.6]	−14.8 (−24.0 to −5.7) [*P* = 0.002]

Abbreviations: CI, confidence interval; NFPA, non-functioning pituitary adenoma; PGWB, psychological general well-being; SD, standard deviation; SEM, standard error of the mean.

* Adjusted for baseline values using analysis of covariance (ANCOVA).

^†^ No. of patients in control and intervention groups, respectively.

^‡^ Includes acromegaly, thyrotropinoma, prolactinoma, craniopharyngioma and Rathke’s cleft cyst.

The impact of the COVID-19 pandemic on total PGWB score was determined in the intervention group for patients who were treated before (*n* = 39) and during the pandemic (*n* = 61). The change in total PGWB score between baseline and 12 months did not differ significantly between these groups, and the mean adjusted difference between the groups was −0.5 (95% CI −7.7 to 6.7; *P* = 0.089).

### Health status, fatigue and general self-efficacy

Secondary endpoints evaluating self-reported health status did not show any statistically significant differences between the control and intervention groups (EQ-5D-5L, MFI-20 and GSE in Table 4).

**Table 4:** Baseline values and adjusted and unadjusted mean differences in health status questionnaire scores from baseline to 12 months.

Health status questionnaire	Baseline values		Mean differences				Adjusted* mean difference between groups (95%CI) [significance]
	Mean (IQR) Median (range)		Control group (*n* = 68)		Intervention group (*n* = 86)		
	Control group (*n* = 68)	Intervention group (*n* = 86)	Mean (SD) Median (range)	Adjusted* mean (SEM) [95% CI]	Mean (SD) Median (range)	Adjusted* mean (SEM) [95% CI]	
EQ-5D-5L	64(23) 70 (45 to 85)	65 (21) 70 (50–80)	6 (21.4) 5 (−68 to 65)	5.2 (2.2) [0.9–9.5]	6.8 (18.9) 5 (−25 to 70)	7.4 (2.0) [3.5–11.3]	−2.2 (−8.1 to 3.7) [*P* = 0.46]
MFI-20							
General fatigue	13 (5) 14 (9–18)	13 (5) 14 (9–18)	−1.7 (3.5) −1 (−10 to 8]	−1.7 (0.5) [−2.7 to 0.8]	−1.8 (4.7) −1 (−15 to 9)	−1.8 (0.4) [−2.7 to 0.9]	−0.1 (−1.3 to 1.5) [*P* = 0.90]
Physical fatigue	12 (5) 13 (8–17)	12 (5) 13 (7–16)	−1.4 (4.3) −1 (−15 to 11)	−1.4 (0.5) [−2.4 to 0.4]	−1.1 (4.6) −1.1 (−15 to 11)	−1.1 (0.4) [−2.0 to 0.2]	−0.3 (−1.6 to 1.1) [*P* = 0.68]
Reduced activity	12 (5) 12 (8–17)	12 (5) 12 (7–16)	−1.6 (4.0) −1 [−11 to 8)	−1.37 (0.5) [−2.4 to 0.4]	−1.3 (4.6) −1 (−16 to 7)	−1.5 (0.4) [−2.3 to 0.6]	0.1 (−1.2 to 1.5) [*P* = 0.85]
Reduced motivation	9 (4) 8 (6–12)	10 (4) 9 (6–12)	−0.9 (3.2) (−9 to 6)	−1.0 (0.4) [−1.8 to −0.2]	−1.6 (3.9) −1 (−12 to 7)	−1.5 (0.3) [−2.2 to 0.9]	0.5 (−0.5 to 1.6) [*P* = 0.31]
Mental fatigue	11 (5) 11 (7–16)	11 (4) 11 (7–15)	−1.4 (3.4) (−8 to 11)	−1.5 (0.4) [−2.3 to 0.7]	−1.1 (3.6) 0 (−11 to 9)	−1.03 (0.4) [−1.7 to 0.3]	−0.5 (−1.6 to 0.6) [*P* = 0.38]
GSE	30 (6) 30 (27–34)	31 (5) 32 (28–35)	1.6 (4.6) −1 (−11 to 13)	1.4 (0.5) [0.4–2.5]	0.8 (4.8) 1 (−17 to 16)	0.9 (0.5) [0–1.9]	0.5 (−0.9 to 2.0) [*P* = 0.46]

Abbreviations: CI, confidence interval; EQ-5D-5L, EuroQoL 5-Dimension 5-Level; GSE, generalized self-efficacy; IQR, interquartile range; MFI-20, Multidimensional Fatigue Inventory-20; SD, standard deviation; SEM, standard error of the mean.

* Adjusted for age, gender, education and cortisol insufficiency using analysis of covariance (ANCOVA).

## Discussion

To our knowledge, this is the first prospective intervention study based on the principles of person-centered care for patients with pituitary tumors. The study showed an improvement in psychological well-being from before surgery to 12 months post-surgery in both the intervention and control groups. Person-centered practice did not show any difference in psychological well-being between the groups, measured as total PGWB score. However, the intervention group reported a reduced level of anxiety compared to the control group 12 months after surgery.

The study evaluated the effect of person-centered practice by means of extended support with a name-given nurse care manager, including an interdisciplinary team as well as peer support during outpatient care after surgery. From our results, we can conclude that standard care was as efficient as our intervention in terms of psychological well-being, self-reported health status and perceived self-efficacy. This might, in fact, reflect that standard care and the intervention possess similar ‘active’ components, engagement and understanding from the healthcare professional as well as continuity within a specialized outpatient clinic. Our specialized center has long been dedicated to the care of patients with pituitary tumors, employing highly skilled personnel at all levels. This pre-existing expertise might explain why the impact of our intervention was smaller than expected.

The improvement in anxiety in the intervention group is important, as anxiety is a highly prevalent symptom reported among patients with pituitary tumors and hypopituitarism (5, 6, 7, 8). A randomized controlled trial that evaluated a patient education program tailored to enhance self-management among patients with pituitary tumors showed an improvement in mood directly after the intervention, but mood had declined again by the 6-month follow-up (13). The same study showed enhanced self-efficacy in the group receiving education, which could not be detected in our study. Improved self-efficacy is a desirable outcome in the management of patients with a long-term illness. Our study could not detect any differences in general self-efficacy, which can be explained by the fact that both the intervention group and the control group reported self-efficacy in line with the general population (22) at baseline of the study. Another initiative to enhance care for patients with NFPAs after surgery aimed to more thoroughly focus on patients’ preferences and needs, along with a focus on alleviation of distressing symptoms and lifestyle changes (23). These two previous studies (13, 23) along with ours adhere to the European standard that stipulates criteria for patient involvement in healthcare to progress toward person-centered care through patient narratives, partnership and documentation (https://www.sis.se/produkter/halso-och-sjukvard/medicin-allmant/halso-och-sjukvardstjanster-allmant/ss-en-173982020/).

The timing of the intervention was chosen based on the importance of the time period following pituitary surgery. This is a period that entails a variety of challenges for patients, such as the adjustment to having a rare disease, a tumor located in the head, a tumor remnant after surgery in need of long-term surveillance and pituitary hormone deficiencies that may need lifelong replacement. Furthermore, patients need to understand, be involved in and adhere to medical and surgical interventions (2, 8).

Although the majority in this study were patients with NFPAs, patients with pituitary tumors comprise a heterogeneous population due to different tumor subtypes, which is also reflected in our study. In our subgroup analysis, we may have identified patients who are more likely to benefit from person-centered practice following surgery. This analysis showed that patients who underwent their first pituitary surgery and received the intervention reported greater improvements in psychological well-being compared to those receiving standard care after their first surgery. Furthermore, patients in the intervention group with pituitary tumors other than the NFPA showed a greater improvement in their psychological well-being than those in the control group. These results suggest that certain subgroups of patients gain more when being cared for within a person-centered practice. However, these results should be interpreted with caution due to the small number of patients in the subgroups. Previous studies on the implementation of person-centered care have demonstrated beneficial effects for specific subgroups of patients, such as the most elderly (24) or those requiring hospitalization for acute care (25). These findings underscore the necessity of critically reviewing each care context to identify patient subgroups that would benefit most from person-centered care.

When evaluating the impact of our intervention, we need to consider that the recovery trajectory after pituitary surgery varies among patients and is influenced by numerous factors. Many patients experience a significant improvement in their visual function and relief from headaches (26). Some patients have an improved pituitary function after surgery, while others develop new endocrine deficiencies that necessitate initiation of endocrine replacement therapies, all of which affect the patients’ health status. All these factors cannot be fully accounted for in an intervention study investigating person-centered practice.

Other aspects that might have influenced the intervention are situational aspects due to the long recruitment time period, as well as the unknown impact of the COVID-19 pandemic, which occurred during the intervention. The analysis we performed to address the impact of the pandemic did not show any significant differences in the intervention group, but there may be aspects of the pandemic that we do not understand. To ensure a sustained implementation of the intervention over time – particularly the role of the nurse care manager – the clinic’s quality and safety improvement nurse and a researcher regularly met with the nurse care managers to provide support and discuss the components of the intervention and its direct integration into patient care. We can conclude that continuity with the interdisciplinary team, including the follow-up time points with the nurse care manager, was maintained in accordance with the protocol, although a majority of the follow-ups with the nurse care manager were changed to distance care during the pandemic. However, more transparent documentation made in the health books and health plans by the patient and the nurse care manager might have deepened the necessity for other health-promoting activities, other needs of support and symptom alleviation (27). To determine exactly which health-promoting interventions were initiated, a review of the medical records would be necessary; however, this was not investigated in this study. In addition, not all patients and relatives were reached by the patient education program and thereby missed out on education and peer support. A limitation in the study is the quasi-experimental design that was decided on based on considerations highlighted as important in the design and outcome in the evaluation of a complex intervention such as person-centered care (28). A randomized controlled trial design was not deemed appropriate, as the person-centered practice requires changes in the entire healthcare setting, which cannot be switched on and off in the interaction between different patients within the same care setting. Moreover, the implementation of person-centered care into clinical practice is a complex process that demands a combination of many different components specifically adapted to the clinical setting to align with the ethics of person-centered care (12, 28, 29). While the study size can be considered large in the context of a prospective study, the heterogeneity of tumor types presents a limitation in interpreting the study’s results.

We conclude that the intervention with the person-centered practice did not result in major advantages in terms of health or psychological well-being, except for reduced anxiety levels. Our study may, however, suggest that certain subgroups of patients derive greater health benefits from a person-centered practice following pituitary surgery. Therefore, the development, improvement and assessment of person-centered care for patients with pituitary tumors should persist by incorporating and evaluating specific health-promoting activities following surgery.

## Supplementary materials



## Declaration of interest

DSO has served as a consultant to Novo Nordisk and Pfizer, has received unrestricted grants from Pfizer, and is an employee of AstraZeneca since 30 August 2021. GJ has served as a consultant for Novo Nordisk and AstraZeneca, has received lecture fees from Novo Nordisk and Pfizer, and has received unrestricted research grants from Novo Nordisk, Pfizer and Shire/Takeda. All of the other authors declare that they have no conflicts of interest.

## Funding

Funding for this study was granted by the Swedish government and the county councils through the ALF-agreement (ALFGBG-966066 and 719531), Cancerfonden (Project grant 211774 Pj) and The Healthcare Board, Västra Götaland Regionhttps://doi.org/10.13039/100007212 (VGFOUREG-929693). Study design, data collection, data analysis, data interpretation and decisions regarding dissemination of the study results did not involve any contribution from the funding sources.

## Patient consent

All participants provided written informed consent before any part of the study was performed.

## Author contribution statement

S Jakobsson (Conceptualization (equal), Investigation (lead), Project administration (lead), Formal analysis (lead), Writing – original draft (lead)), Writing review & editing (equal)), O Ragnarsson (Conceptualization (equal), Investigation (lead), Project administration (equal), Formal analysis (lead), Writing review & editing (equal)), T Hallén (Conceptualization (equal), Investigation (equal), Project administration (equal), Formal analysis (support), Writing review & editing (equal)), D Krabbe (Conceptualization (support), Investigation (support), Project administration (support), Writing review & editing (equal)), A-C Olofsson (Conceptualization (equal), Investigation (support), Project administration (lead), Writing review & editing (support), D S Olsson (Conceptualization (equal), Investigation (equal), Project administration (support), Formal analysis (equal), Writing review & editing (equal)), P Trimpou (Conceptualization (support), Investigation (support), Writing review & editing (equal)), T Skoglund (Conceptualization (equal), Investigation (lead), Project administration (equal), Formal analysis (equal), Writing review & editing (equal)), Funding acquisition (lead), G Johannsson (Conceptualization (equal), Investigation (lead), Project administration (equal), Formal analysis (lead), Writing – original draft (lead)), Writing review & editing (equal)), Funding acquisition (lead).

## Data availability

All data relevant for this publication are available in the manuscript. The self-reported questionnaires and responses are in Swedish. Upon reasonable request, data can be made available in consideration of research ethical aspects, EU data protection act and Swedish data protection act.

## Institutional review board statement

The regional ethical review board in Gothenburg, Sweden, approved the study protocol (approval reference 387-15).
