# Supplementary material for: Person-centered support for patients with a pituitary tumor following surgery
Source: Endocr Connect. 2025 Jan 28;14(3):e240686. doi: 10.1530/EC-24-0686 (PMC11799750; doi:10.1530/EC-24-0686)
Supplement: Supplementary file 1 [file supplementary_materials.pdf]

SUPPLEMENTARY MATERIAL

Person-centered support for patients with a pituitary tumor following surgery
by Sofie Jakobsson, Oskar Ragnarsson, Tobias Hallén, David Krabbe, Ann-Charlotte Olofsson, Daniel S. Olsson, Penelope Trimpou, Thomas Skoglund, and Gudmundur Johannsson

Table S1. Patient characteristics and clinical data in the control group receiving standard care and the interventional group receiving person-centered practice.

	**Control group (*n* = 83)**	**Interventional group (*n* = 100)**
Gender, *n* (%)		
Women	39 (47)	42 (42)
Men	44 (53)	58 (58)
Age, median (range), years	57 (19–85)	58 (24–87)
Living arrangements, *n* (%)		
Cohabitating	59 (72)	70 (73)
Living alone	23 (28)	26 (27)
Missing	1	4
Country of birth, *n* (%)		
Sweden	69 (84)	83 (86)
Outside Sweden	13 (16)	13 (14)
Missing	1	4
Education, *n* (%)		
Elementary school	18 (22)	12 (13)
High school	36 (44)	53 (55)
University	28 (34)	31 (32)
Missing	1	4
Occupation, *n* (%)		
Working/studying	38 (48)	51 (53)
Other	41 (52)	45 (47)
Missing	4	4
Diagnosis, *n* (%)		
NFPA	55 (66)	72 (72)
Cushing's disease	5 (6)	8 (8)
Acromegaly	10 (12)	13 (13)
Thyrotropinoma	1 (1)	1 (1)
Prolactinoma	2 (2)	0
Craniopharyngioma	3 (4)	2 (2)
Rathke's cleft cyst	4 (5)	2 (2)
Other*	3 (4)	2 (2)
Cortisol insufficiency†, *n* (%)		
Yes	32 (39)	33 (33)
No	51 (61)	67 (67)
Prior surgery, *n* (%)		
Yes	15 (18)	14 (14)
No	68 (82)	86 (86)
Surgery type, *n* (%)		
Acute	12 (14)	7 (7)
Planned	71 (86)	93 (93)

*Includes chondroid chordoma, hypophysitis, pituitary cytoma, and unclear (each *n* = 1).
†Post-surgery. Abbreviation: NFPA, non-functioning pituitary adenoma.

SUPPLEMENTARY MATERIAL

Person-centered support for patients with a pituitary tumor following surgery by Sofie Jakobsson, Oskar Ragnarsson, Tobias Hallén, David Krabbe, Ann-Charlotte Olofsson, Daniel S. Olsson, Penelope Trimpou, Thomas Skoglund, and Gudmundur Johannsson

Table S2. PGWB total and subscale scores for control and intervention groups.

PGWB total or subscale scores	Group	Mean (SD)			
		Before surgery	At discharge	Follow-up at 3–6 months post-surgery	Follow-up at 12 months post-surgery
Total	Control	90 (23)	97 (20)	102 (18)	99 (19)
	Intervention	91 (21)	92 (20)	101 (20)	102 (19)
Subscale					
Anxiety	Control	21 (6)	23 (6)	24 (5)	23 (5)
	Intervention	21 (6)	22 (5)	24 (5)	25 (5)
Depression	Control	14 (3)	16 (2)	16 (2)	15 (3)
	Intervention	14 (3)	15 (3)	16 (3)	16 (3)
Positive well-being	Control	15 (4)	15 (4)	16 (4)	16 (4)
	Intervention	15 (4)	14 (4)	16 (4)	16 (4)
Self-control	Control	14 (4)	15 (3)	15 (3)	15 (3)
	Intervention	14 (3)	15 (3)	15 (3)	15 (3)
General health	Control	12 (3)	12 (3)	14 (3)	13 (3)
	Intervention	12 (4)	11 (3)	14 (3)	14 (3)
Vitality	Control	14 (5)	15 (5)	16 (5)	16 (5)
	Intervention	14 (5)	14 (5)	16 (5)	16 (5)

Total score range 22–132. Subscales ranges: anxiety (5–30), depression (3–18), positive well-being (4–24), self-control (3–18), general health (3–18), and vitality (4–24). Higher scores indicate better psychological well-being. Abbreviation: PGWB, Psychological General Well-Being scale.
